# Dysmotility in Esophageal Atresia: Pathophysiology, Characterization, and Treatment

**DOI:** 10.3389/fped.2017.00130

**Published:** 2017-05-31

**Authors:** Christophe Faure, Franziska Righini Grunder

**Affiliations:** ^1^Esophageal Atresia Clinic, CHU Sainte-Justine, Montreal, QC, Canada

**Keywords:** esophageal motility disorders, gastroesophageal reflux, aspiration, dysphagia, feeding disorders, high-resolution esophageal manometry, impedancemetry

## Abstract

Esophageal dysmotility is almost universal after esophageal atresia (EA) repair and is mainly related to the developmental anomaly of the esophagus. Esophageal dysmotility is involved in the pathophysiology of numerous symptoms and comorbidities associated with EA such as gastroesophageal reflux disease, aspiration and respiratory complications, and symptoms of dysphagia and feeding disorders. High-resolution esophageal manometry (HREM) has facilitated the characterization of the dysmotility, but there is an incomplete correlation between symptoms and manometrical patterns. Impedance coupled to HREM should help to predict the clinical outcome and therefore personalize patient management. Nowadays, the management of esophageal dysmotility in patients with EA is essentially based on treatment of associated inflammation related to peptic or eosinophilic esophagitis.

Following esophageal atresia (EA) repair, motility disorders of the esophagus are almost universal and may lead to gastroesophageal reflux (GER), aspiration, feeding disorders, and dysphagia in the first few months and years of life. Later on, chronic acid exposure of the esophageal mucosa due to abnormal esophageal motility can lead to Barrett’s esophagus and esophageal carcinoma, which are a major concern (1). In this review, we will focus on the definition, pathophysiology, and treatment of esophageal dysmotility in patients operated for EA.

## The Burden of Esophageal Dysmotility after EA Repair

In patients operated for EA, abnormal motility of the esophagus remains the key pathophysiological catalyst leading to digestive and respiratory morbidity throughout life. Indeed, esophageal motility is involved not only in the process of transporting food from the mouth to the stomach but also plays a central role in the defense of the esophagus against gastric reflux. Furthermore, a well-organized swallowing process, from the mouth to the esophagus guarantees an adequate protection of the respiratory tract against aspiration. The following section highlights the consequences of the impaired esophageal motility in patients with EA.

### Esophageal Dysmotility and GER

After EA repair, GER is highly prevalent from birth to adulthood. A recent review reports that 22–63% of patients are affected by GER (1). Complications such as peptic esophagitis, peptic strictures, worsening of anastomotic strictures, gastric and intestinal metaplasia of the esophageal mucosa, and even esophageal adenocarcinoma have been described in EA patients, thereby highlighting the severity of the GER in this population (1). EA patients likely develop a severe GER for various reasons including anatomical anomalies (hiatal hernia, abnormal position of the intrathoracic part of esophagus), vagal nerve surgical injury with abnormal gastric emptying and esophageal dysmotility. The latter leads to abnormal esophageal clearance, which increases the duration of mucosal exposure to gastric juice and acid. Several authors have shown in children and in adults that the greater the degree of esophageal dysmotility, the more the GER is complicated by epithelial metaplasia suggesting a correlation between motor disturbances and severity of reflux (2–4).

### Esophageal Dysmotility and Dysphagia

Dysphagia as a symptom is reported in a majority of patients with EA even though most patients learn to adapt to their unique anatomical and physiological state and do not report any complaints. Studies have reported that dysphagia occurs in 21–84% of patients with EA at all ages after surgical repair (2, 4–7). A recent review reports a prevalence of more than 50% in patients older than 10 years (8). Symptoms of dysphagia are not specific and vary according to the age of the patient and whether or not solid food has been introduced. Dysphagia should be evoked in patients with EA who present with food aversion, food impaction, difficulty in swallowing, odynophagia, choking, cough, pneumonia, alteration in eating habits, vomiting, and malnutrition (1). Children may have occasional difficulties with swallowing, are reported as sloweaters or excessive drinkers during meals. Up to three of four of patients with dysphagia report significant changes in their eating habits (need to drink, change in diet, last to finish meal) (2). The etiology of the dysphagia may include inflammatory (peptic or eosinophilic esophagitis) and anatomic causes (anastomotic stricture, congenital stenosis, peptic stricture, post-fundoplication obstruction, vascular compression, anastomotic diverticulum, or mucosal bridge), and abnormal esophageal motility (1). Dysphagia therefore warrants a systematic workup to rule out all of the abovementioned etiologies. In the absence of one of the previously outlined causes, esophageal dysmotility, which impairs a normal bolus transit, remains the most likely explanation (1).

### Esophageal Dysmotility As a Risk Factor for Aspiration and Feeding Disorders

Abnormal esophageal motility, thereby hampering an adequate coordination between aerial and digestive tracts, may also foster feeding disorders and aspiration during swallowing, with extraesophageal complications such as recurrent pneumonia, bronchitis, or chronic cough. Once again many hypotheses such as anastomotic stricture, congenital esophageal stenosis, recurrent or missed fistulae, laryngeal cleft, or developmental issues must be carefully ruled out. If the workup is negative, the motor disturbance of the esophagus remains the explanation. The esophageal dysmotility may involve upper esophageal sphincter (UES) dynamics (9, 10) and/or abnormal bolus clearance leading to secretions or food retention in the proximal pouch or distal esophagus or an esophageal pooling over a fundoplication.

## Characterization of Esophageal Dysmotility

Esophageal motility has been assessed in children and adults with EA by esophageal manometry [water perfused (4, 11–16) or high resolution (2, 3, 7, 17–19)], impedancemetry (19, 20), or videofluoroscopy (21, 22). Studies have reported anomalies at each level of the esophagus including larynx and vocal cords (23–25) and gastric motor function (15, 26).

### Upper Esophageal Sphincter

The UES function has been reported to be normal by most authors (2, 7), but incomplete relaxation has been described in newborns (27). When evaluated by videomanometry, an inadequate coordination between pharyngeal contraction and UES relaxation was found in adults (21). Aspiration during swallowing assessed by videofluoroscopy has been reported in 20–47% of children with EA (9, 10).

### Esophageal Peristalsis

**Figure 1:**
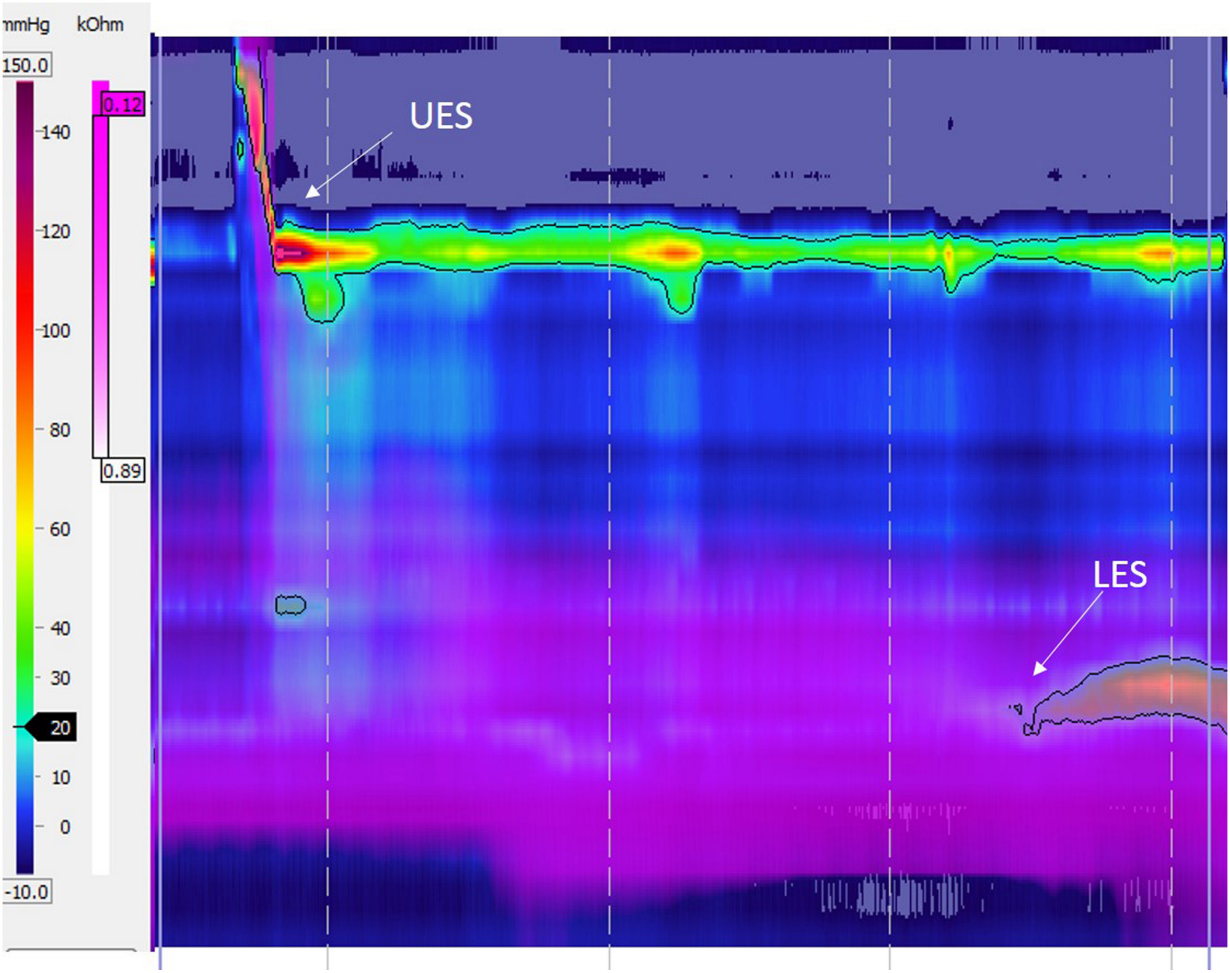
High-resolution esophageal manometry tracing recorded in a patient with type C esophageal atresia: normal upper esophageal sphincter (UES), pattern of aperistalsis, and normal lower esophageal sphincter (LES) pressure and relaxation. The purple color displays intraesophageal impedance variations after a liquid swallow. Note that the bolus clearance is not complete with residual liquid in the distal esophagus.

**Figure 2:**
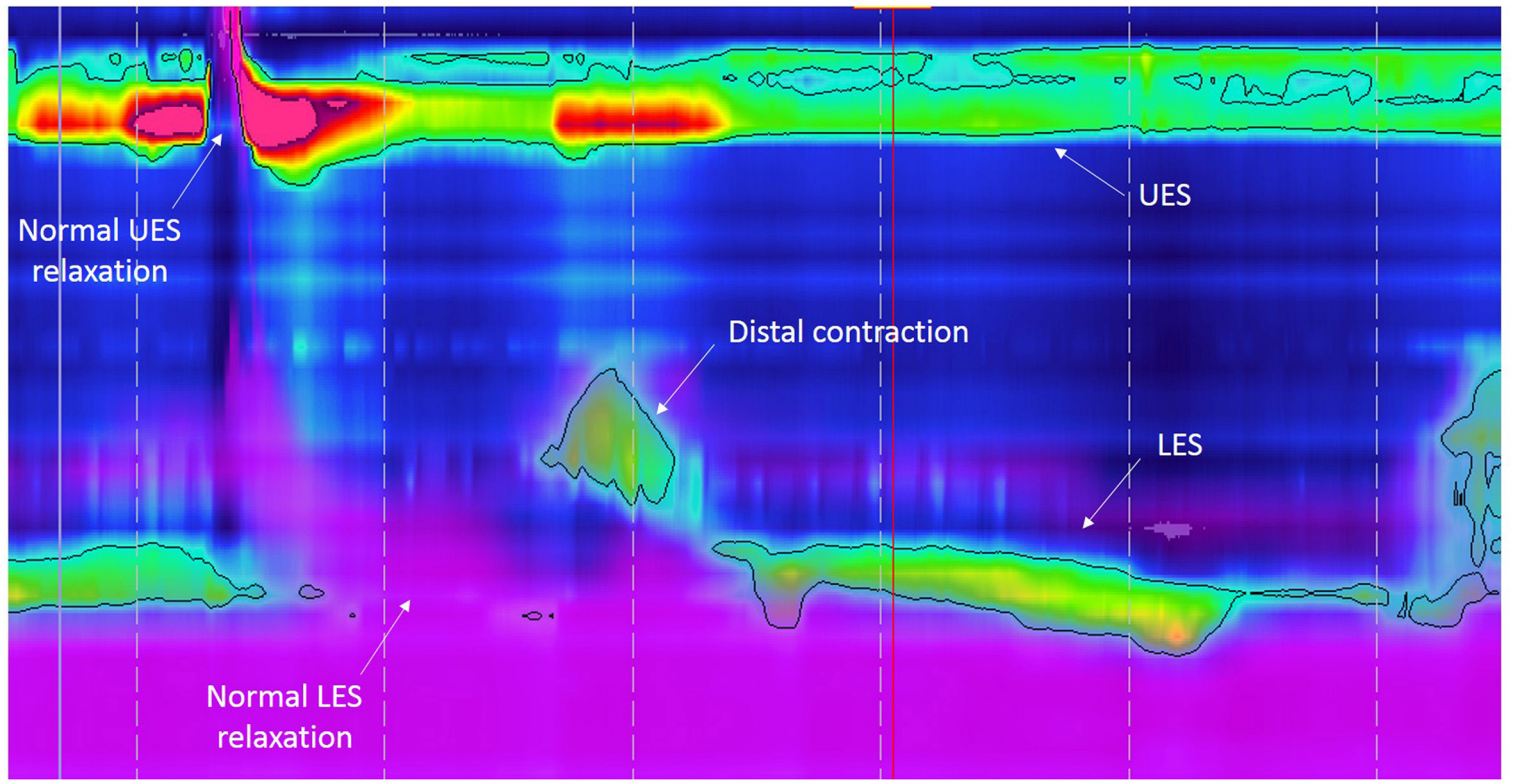
High-resolution esophageal manometry tracing recorded in a patient with type C esophageal atresia: normal upper esophageal sphincter (UES), pattern of distal contraction, and normal lower esophageal sphincter (LES) pressure and relaxation. The purple color displays intraesophageal impedance variations after a liquid swallow. Note that the bolus clearance is almost complete with very few residual liquid in the esophageal body.

Abnormal esophageal peristalsis has been reported in almost all patients with EA. It is found in children (2, 3, 7, 14, 15, 17, 27–30) and persist throughout life as demonstrated by adult studies (4, 11–13, 15, 16). Esophageal dysmotility in EA was recently described using high-resolution manometry (HREM) with three types of abnormalities observed: aperistalsis (Figure 1), isolated distal contractions (Figure 2), and pressurization (2, 3, 19). GER-related symptoms are prominent in patients with aperistaltic esophagus (2, 3). Type A and long gap defect seem to have a more severe esophageal motor function than type C (2). Manometrical abnormalities are significantly worse in those with epithelial metaplasia (4). Interestingly, correlation between symptoms of dysphagia, motility abnormalities, and bolus transit is imperfect. Impedance associated with high-resolution manometry permits to correlate the degree of motility abnormalities with bolus transit (31).

### Lower Esophageal Sphincter (LES)

In almost all studies including those using HREM, LES pressure, and function are similar to controls (2, 7, 12, 27, 28, 32, 33). A study conducted in children with non-complicated type C EA shows that transient LES relaxation is the pathophysiological mechanism in two of three of the reflux episodes (15). However, no data on transient LES relaxation are available in long gap EA, and the latter results may not be applied to patients with high-tension anastomosis leading to abnormal anatomic location of the LES as well as highly impaired esophageal body motility.

## Etiology of the Esophageal Dysmotility

The etiology of the esophageal dysmotility remains controversial. It may be related to (1) factors due to abnormal development of the esophageal smooth muscle and intrinsic innervation and of the vagus nerve or (2) to factors associated with surgical techniques, fibrotic scars, and postoperative complications. Data indicating that the congenital malformative process plays a major role are prominent in the literature, although surgical repair may exacerbate the esophageal dysmotility.

### Primary Motility Disorder

Pathological data are supportive of the role of abnormal intrinsic and vagal innervation of the esophagus. Analysis of esophageal innervation in dead EA newborn has reported abnormalities in the Auerbach plexus (plexus hypoplasia and abnormal interganglionic network) (34). Other studies have also reported hypoplasia of esophageal innervation or smooth muscle (35, 36) or interstitial cells of Cajal (37) in the proximal pouch (36, 38, 39), distal esophagus (36, 37, 39, 40), or in the fistula (35, 41). Animal studies in a rat adriamycin model of EA have similarly shown abnormal vagal and intrinsic innervation of the esophagus (36, 42). Esophageal manometry performed prior to surgery in 20 newborns with EA demonstrated motor abnormalities in the proximal (pouch) and distal esophagus (27). Likewise, abnormal esophageal motility patterns have been reported in children and adults with isolated TEF without atresia before surgical repair (43, 44) suggesting that abnormal development of the esophagus has consequences on the esophageal motility function.

### Secondary Motility Disorder

The dysmotility may also be secondary to the dissection during surgery, which can damage the vagal nerve and its esophageal branches as shown by Davies in autopsied newborns with EA (40). Therefore, the operative dissection may also likely worsen the dysmotility.

## Treatment

There is no controlled study on prokinetic drugs for treatment of esophageal dysmotility associated with EA. Since esophageal muscle and innervation are deficient and since the anastomotic zone is fibrotic, the efficacy of such drugs is unlikely to be significant especially in those patients with aperistalsis. However, in patients with remnants of distal peristalsis, one could expect some benefit with prokinetic medications, but objective data are lacking. Cisapride, a 5HT4 agonist, and bethanechol, a cholinergic agonist, are supposed to enhance esophageal motility. Cisapride has been reported to increase amplitude of esophageal peristalsis (45), but its availability is restricted due to risk of prolonged QT interval and severe cardiac arrhythmia. Bethanechol acts on muscarinic receptors of the smooth muscle and thereby increases esophageal contractions and clearance (46). However, cholinergic side effects (bronchial constriction) limit its use in asthmatics. Baclofen inhibits the transient LES relaxations (47) and can be used for treatment of GER. Its use may be limited due to side effects (dizziness). Metoclopramide, domperidone, and erythromycin act on gastric emptying (48).

Treatment of acidic GER by PPIs or H2-receptor antagonists is mandatory as well as careful screening and treatment of eosinophilic esophagitis (topical corticosteroids and allergen withdrawal), which are aggravating factors for the esophageal mucosa with significant impact on esophageal motility (49–51) and esophageal wall compliance.

## Unanswered Questions

Even though esophageal dysmotility has been reported in infants, toddlers, children, adolescents, and adults, the natural history of esophageal dysmotility in patient with EA is unknown since no prospective longitudinal study has been conducted thus far. The implementation of such a study would be extremely difficult for ethical reasons given the invasiveness of the techniques used for assessing esophageal motility.

The introduction of high-resolution manometry coupled with esophageal impedance should lead to a better understanding of the relationship between esophageal dysmotility, bolus clearance, and symptoms as well as clinical outcome and especially long-term complications such as esophageal metaplasia, Barrett esophagus, and cancer. A new method, the pressure-flow analysis (PFA), to analyze and measure esophageal motility and its effects on bolus clearance has been recently made available (52). PFA, by quantifying the interactions between bolus transport and pressure generation, may help in further investigating these patients. Validation and application of this method in EA patients are warranted and ongoing.

Anomalies of sensory function have not been studied even though sensory innervation is as affected as the motor nerves in EA. One study using the acid perfusion test conducted in adult EA patients with erosive esophagitis reports an absence of sensation in 11 of 14 patients suggesting an impairment of the visceral esophageal sensitivity (11). Pharyngeal sensitivity and esophageal sensitivity play an important role in swallowing and feeding processes, as well as in the perception of symptoms.

Tissue engineering of injured or fibrotic esophagus could ultimately lead to recovery of normal esophageal motility. On the other hand, the attempts to generate engineered tissues must carefully take into account the importance of all components of the esophageal wall involved to generate a neo-esophagus with normal peristalsis and sphincter function.

## Summary

Esophageal dysmotility is almost universal after EA repair and is mainly related to the developmental anomaly of the esophagus. Esophageal dysmotility is involved in the pathophysiology of numerous symptoms and comorbidities associated with EA such as GER disease, aspiration and respiratory complications, and symptoms of dysphagia and feeding disorders. High-resolution esophageal manometry (HREM) has facilitated the characterization of the dysmotility, but there is an incomplete correlation between symptoms and manometrical patterns. Impedance coupled to HREM should help to predict the clinical outcome and therefore personalize patient management. Nowadays, the management of esophageal dysmotility in patients with EA is essentially based on treatment of associated inflammation related to peptic or eosinophilic esophagitis.

## Author Contributions

CF and FG wrote the draft. CF finalized the manuscript.

## Conflict of Interest Statement

The authors declare that the research was conducted in the absence of any commercial or financial relationships that could be construed as a potential conflict of interest.
